# Supplementary material for: Are carbon emissions trading and green financial instruments synergistic? -Comprehensive quantitative research based on content analysis
Source: PLoS One. 2024 Mar 7;19(3):e0298601. doi: 10.1371/journal.pone.0298601 (PMC10919601; doi:10.1371/journal.pone.0298601)
Supplement: S1 Data — (ZIP) [file pone.0298601.s001.zip › Data/地级市Z_F工作报告.zip!/╣π╬≈/░┘╔1⁄2╩╨/└┤╫╘╣1⁄2╓┌║┼í╛┬φ┐╦╩2╛▌═°í┐_2017.docx]

一、2016年工作回顾

2016年是我市决战贫困决胜小康的起步之年。面对错综复杂的宏观环境和持续加大的经济下行压力，我们坚决贯彻落实中央、自治区和市委决策部署，牢固树立和践行新发展理念，全力以赴稳增长、促改革、调结构、惠民生、防风险，以脱贫攻坚统领全市经济社会发展，扎实推进供给侧结构性改革，经济运行总体平稳、稳中向好、逆势上扬，社会和谐稳定，民族团结和睦，边疆巩固安宁，人民安居乐业，实现了“十三五”良好开局。

全市地区生产总值1114.31亿元，增长8.8%，其中一产增长3.9%，二产增长9.6%，三产增长10.4%；规模以上工业增加值482.91亿元，增长9.7%；固定资产投资1061.4亿元，增长3.9%；财政收入123.22亿元，增长7.6%；社会消费品零售总额246.84亿元，增长11.6%；外贸进出口总额138亿元，增长36.4%；银行业金融机构各项存贷款总额1928.1亿元，增长16%。

——打赢脱贫攻坚第一仗。田东县、右江区和田阳县成功“摘帽”。全市实现21.84万贫困人口脱贫、134个贫困村“出列”，贫困发生率由20.5%下降到14.66%，超额完成自治区年初下达的脱贫目标任务。全国产业扶贫现场会、全区“一帮一联”现场会在百色召开，“三双”工作法在全区推广，芒果产业成为全国首个以特色产业带动精准脱贫的范例。百色精准脱贫经验得到中央、自治区肯定。

——多项经济指标提速晋位。地区生产总值首次突破1000亿元，成为全区第6个GDP跻身“千亿元俱乐部”的地级市，增速排全区第3位，比上年提升4位，取得自2004年以来在全区最好排位，分别高于全国、全区2.1个百分点和1.5个百分点。其中，一产增速排全区第4位；二产增速排全区第5位，比上年提升2位；三产增速排全区第2位，比上年提升4位。规模以上工业增加值增速排全区第5位，比上年提升3位。财政收入增速排全区第6位，比上年提升4位。社会消费品零售总额增速排全区第2位，比上年提升3位。外贸进出口总额增速排全区第2位，排全区口岸城市第1位。银行业金融机构各项存贷款总额排全区第5位，增速排全区第2位，比上年提升9位。

——多项经济结构优化凸显。第三产业对经济增长的贡献率达35.8%，比上年提高1.5个百分点，拉动经济增长3.2个百分点。铝产业结构进一步优化，氧化铝、电解铝、铝材加工三项产量比由2015年的26.4 : 1 : 6.6转变为2016年的16.8 : 1 : 4.9，电解铝、铝材加工分别增长68.4%和21.3%。铝加工由建筑型材逐步向汽车轮毂、发动机缸盖等高附加值产品发展。

——多项发展新动能累积迸发。区域电网一期工程竣工投产，每吨电解铝生产用电成本下降2000元左右，促进新增工业产值50亿元。全国政策性金融扶贫实验示范区创建取得新进展，成立扶贫投融资平台公司26家，投放扶贫信贷35亿元。左右江老区振兴发展基金、城镇化发展基金、扶贫开发基金、金砖城市发展基金等平台融资规模达560亿元。银行业金融机构新增贷款突破100亿元。

——多项重大项目竣工投入使用。百色干部学院建成，标志着我市加强干部教育阵地建设取得新进展、培训基地体系得到新完善、培训能力建设得到新提升，成为全区干部教育的“红色殿堂”，为百色对外宣传添加了一张崭新靓丽的名片。南昆高铁百色至昆明段正式开通运营；百色市图书馆竣工并对外开放。

——多项工作获得国家乃至国际批复授名。百色水库灌区获批准入国家节水供水重大水利工程项目，龙邦口岸获国家质检总局批复为进境水果指定口岸。百色荣获“全国绿化先进集体”称号。乐业县荣获联合国相关机构“世界长寿之乡”称号。百色起义纪念公园通过国家5A级旅游景区景观质量评审，新增百色芒果、西林火姜、乐业猕猴桃、德保脐橙等4个国家地理标志保护产品，总量达11个，居全区第1位。

——多项民生福祉持续改善。全市财政民生支出281.48亿元，占一般公共预算支出的82.72%。全市60.57万城乡低保对象月平均补助分别为300元和140元，0至3公里的9.79万边境居民月平均补助130元，1318名孤儿享受基本生活补助。机关事业单位和企业退休人员月人均养老金增长7.2%。城乡居民基本养老保险月人均养老金达92元。城镇新增就业2.12万人，农村劳动力转移新增就业5.4万人次，城镇登记失业率2.89%。城镇居民人均可支配收入26919元，增长7.5%。农村居民人均可支配收入9348元，增长10.6%，增速排全区第1位。群众获得感不断提升。

一年来，主要开展了以下工作：

一是以脱贫攻坚统领全局，精准脱贫首战告捷。扎实开展“八个一批”“十大行动”和“十个到村到户”，全市整合投入各类财政扶贫专项资金61.56亿元，是2015年的20倍。20户以上自然屯道路建成3521公里，行政村通村道路硬化完成344个；住房保障解决12905户；人畜饮水项目完成1168个，解决饮水问题4.5万户；366个贫困村的公共服务设施得到完善。易地扶贫搬迁点开工建设102个，完成搬迁入住40244人。金融扶贫贷款余额118亿元，增长98.87%，增量、增速均排全区第1位；累计获得小额信贷贫困户6.34万户，发放扶贫贷款49.32亿元；资产收益扶贫投入6.9亿元，受益贫困户2.19万户。动员419个企业投入2.5亿元资金，帮扶403个贫困村。全面启动与深圳市扶贫协作，获得财政对口帮扶资金1亿元。环球集团在百色成立全区首个民营企业扶贫办。争取世界银行支持平果县、田东县、乐业县、田林县扶贫产业发展资金4.5亿元。累计筹集教育助学捐款9500万元。获得龙光集团捐赠资金1亿元。

二是大力推进结构调整，特色优势产业加速壮大。抓好铝“二次创业”，百矿、苏源等煤电铝一体化项目投产，华磊年产40万吨电解铝及3台35万千瓦自备电厂项目开工建设，一批新的铝产业项目加快布局。百矿集团、华银铝业、平铝集团、强强碳素等4家企业进入中国铝工业百强，全市铝工业百强企业达8家。全市工业园区由15个增加到17个，德保工业集中区产值突破100亿元大关，过百亿元工业园区增加到6个。A类以上工业园区总产值突破1000亿元，达1110亿元，增长13.2%。扎实推进百万亩芒果、百万亩柑桔、百万亩桑蚕、百万亩油茶、“田七回家”工程。蔬菜、水果、蚕茧、烤烟、木材以及肉类、水产品产量保持稳定增长。建成3个自治区级、9个市级、13个县级和9个乡级现代特色农业（核心）示范区。成功举办第九届中国-东盟（百色）现代农业展示交易会。新增市级重点农业龙头企业17家、农民专业合作社1182家、家庭农场192家。凌云浩坤湖、田州古城、德保矮马王国等一批景区成为旅游新名片，乡村旅游、文化旅游、体育旅游日益活跃，全市旅游接待总人数2725万人次，增长16.96%，旅游消费261.69亿元，增长29.63%。靖西龙邦万生隆国际商贸物流中心、中国-东盟农产品交易中心、百色快递物流园区等服务业集聚区建设加快推进，建成了汽车销售一站式服务中心。与浪潮集团、大华智城、视虎科技等企业合作项目加快落地，建成中国-东盟商务信息港项目一期工程。淘宝特色中国百色馆、京东商城百色馆、一号店百色馆、微商百色馆等入驻商家240家，上线产品200多个。电子商务、大数据、云计算及碳酸钙、健康养生等新产业新业态正加快形成。新增“四上企业”201家。

三是供给侧结构性改革精准发力，改革创新扎实推进。淘汰落后铁合金产能2.22万吨，退出煤炭产能59万吨。存量商品房面积同比减少55万平方米，消化周期缩短至13.5个月。银行业金融机构新增贷款增长14.53%，市属重点国有企业融资总额达120.13亿元。落实中小企业“惠企贷”贷款5.79亿元。出台降成本措施43条，帮助企业减负9.8亿元。深化“放管服”改革，取消调整行政审批事项53项，全面公开政府部门权力清单和责任清单。全面推进实施“六证合一、一照一码”登记制度改革，个体工商户“两证整合”，有序推进电子营业执照和全程电子化工作。农村土地承包经营权、农民住房财产权抵押贷款全国试点稳步推进。农村金融改革进一步深化。沿边金融改革取得新进展，跨境人民币业务结算量达70.6亿元，增长106%，引入境外资本融资10.51亿元。国有林场改革扎实推进，田林县国有林场改革经验在全区推广。供销社改革卓有成效。百色获批设立广西高新区，并申报国家级高新区。田东县、平果县、凌云县获批建设广西农业科技园区。百色矿山机械厂、平果锋华科技、广西阿哩哩养生科技等3家企业通过国家高新技术企业认定，全市高新技术企业达17家。工业完成技术创新项目128项，获批自治区级企业技术中心4家，华银铝业有限公司、八桂凌云茶业有限公司获广西“先进质量管理方法标杆企业”称号，5个新产品新技术研发项目通过自治区验收。

四是加快门户经济发展，开放合作不断扩大。加快沿边开发开放重点试验区、边境经济合作区以及跨境经济合作等开发合作平台的规划和申报，推进百色-文山、百色-黔西南州跨省经济合作园区建设。沿边开发开放互联互通、合作平台建设取得新进展。建成靖西万生隆国际商贸物流中心一期工程、新兴边民互市点等开放合作新平台，平孟口岸升级为国家一类口岸，岳圩边民互市点开通。深入开展桂粤合作驻点招商、长三角城市合作项目对接，举办中国-东盟博览会百色产业投资推介会、百色芒果全国推介会等系列活动。全市新签约项目160个，招商引资实际到位资金406.71亿元，增长10.3%。

五是加快推进新型城镇化，城乡基础设施持续改善。加快实施“大县城”战略，平果、靖西列入国家新型城镇化综合试点。百镇建设示范工程取得阶段性成果。百色经靖西至崇左、那坡，联接北部湾和云南沿边高速公路全线贯通，河池至百色、乐业至百色、靖西至龙邦高速公路建设加快。南昆铁路新增二线加快推进。百东新区路网框架显现雏形，南百高速公路百东互通投入使用，百色大道二期工程进展顺利，右江-田阳一体化进程加快。中心城区“美丽右江工程”、棚户区改造取得新成效，龙景区和迎龙区路网、污水管网、绿化亮化等市政基础设施得到进一步改善，高铁站站前广场投入使用。“美丽百色”乡村建设步伐加快。生态乡村建设各项任务目标基本完成，绿化村屯数8295个。宜居乡村建设全面启动。完成饮水安全工程855处。行政村道路硬化率达100%。开通客车线路行政村1575个，通达率87.69%。全市建成区面积新增7.63平方公里，常住人口城镇化率提高到35.5%。

六是加强环境保护和生态建设，绿色发展质量进一步提升。扎实推进国家和自治区对我市121个减排项目、270个环保违规项目和256个信访投诉问题的核算验收和梳理整改，通过中央环保督察。抓好40家国控重点减排企业及重点行业企业减排项目运行监控。强化城市扬尘专项治理。推广使用新能源、清洁能源汽车。继续推进中心城区及各县城区、重点乡镇污水处理设施建设，消除污水直排口。主要污染物排放量同比下降，PM10、PM2.5浓度控制在自治区年度指标内，空气质量优良率达95.4%。实施土地整治15068公顷。植树造林面积达63.1万亩，连续六年排全区第1位，森林覆盖率达67.4%。德保县、那坡县、西林县新增列入国家级重点生态功能区。“国家森林城市”创建扎实推进。完成全市1000人以上农村集中式饮用水水源地保护区划分。市、县级饮用水源地水质达标率达100%。

七是大力补齐民生短板，各项社会事业全面发展。投入财政资金60亿元，实施了十大为民办实事工程。开工建设棚户区改造住房14805套，分配入住7301套，新增发放补贴1595户。实施农村危房改造2.36万户。实施中心城区教育、卫生基础设施建设攻坚战。深入实施学前教育三年行动计划，新建、改扩建幼儿园36所。德保县、田阳县成功通过义务教育均衡发展国家级评估验收。全市义务教育巩固率达92.63%。靖西市、田东县、德保县县级中专综合改革工作获自治区表彰。大力推进卫生计生文化体育事业发展，县级公立医院核定总床位新增3197张。实行新农合大病保险市级统筹，全市新农合参合率99.29%。人口计生目标管理责任制综合考评进入全区前三位。建设村级公共服务中心项目140个。《百色历史通稿》等一批文化作品荣获广西社会科学优秀成果奖。《百色扶贫开发史》等编撰完成。举办了百色市第四届文艺汇演、第四届运动会、第二届残运会和国际山地户外运动挑战赛、全国徒步大会、中国-东盟自然水域垂钓大赛、全国龙舟邀请赛等系列文体活动。全民健身运动深入开展。

应急管理、安全生产、食品安全、反恐维稳、民族团结各项工作开展良好。国防后备力量建设和双拥共建取得新成果。信访、调处、宗教、侨务、保密、税务、人事人才、水库移民、消防、统计、供销、粮食、检验检疫、通信、海关、海事、气象、邮政、地震、农机各项工作取得新成绩。哲学社会科学、新闻出版、决策咨询、地方志、档案、法律援助、妇女儿童、老龄、残疾人等事业取得新进步。工会、共青团、妇联、红十字会、科协、文联等群团组织在经济社会发展中发挥了重要作用。

全面推进依法行政，不断加强政府自身建设。深入开展“两学一做”学习教育，坚决落实中央八项规定和国务院“约法三章”，深入开展懒政怠政专项整治。认真执行市人大及其常委会的决议、决定，自觉接受人大依法监督、政协民主监督和社会监督，广泛听取各民主党派、工商联、无党派人士意见，人大代表议案、建议和政协委员提案办结率100%。完善政府重大决策程序和机制，进一步规范政府权力运行。全面建立政府法律顾问制度，法治政府建设取得新成效。全面推进公车改革。公共资源交易平台运行进一步规范，政务公开和政府信息公开更加透明。审计监督力度加大。绩效管理进一步加强。廉政建设和反腐败斗争取得新成效。

二、2017年工作的总体要求和目标任务

2017年工作的总体要求是：全面贯彻党的十八大和十八届三中、四中、五中、六中全会精神，深入贯彻习近平总书记系列重要讲话精神和治国理政新理念新思想新战略，紧紧围绕自治区第十一次党代会和市第四次党代会重要部署，统筹推进“五位一体”总体布局，协调推进“四个全面”战略布局，坚持稳中求进总基调，把握引领经济发展新常态，以供给侧结构性改革为主线，以脱贫攻坚为统领，坚持“四个优先”发展战略，打造“三中心两区一市”，建设左右江革命老区核心城市，全力保发展、促改革、惠民生、强保障，确保经济平稳健康发展、社会和谐稳定，以优异的成绩迎接党的十九大胜利召开。

经济社会发展主要预期目标是：实现17.5万以上贫困人口脱贫，215个贫困村脱贫“出列”；地区生产总值增长10%以上，财政收入增长7.5%，规模以上工业增加值增长12%，固定资产投资增长13.5%；社会消费品零售总额增长11.5%；进出口总额增长20%；城镇居民、农村居民人均可支配收入分别增长8%、11%；城镇新增就业1.9万人以上，城镇登记失业率控制在4%以内。居民消费价格指数涨幅控制在3.5%以内。节能减排控制在自治区下达的目标以内。

实现今年的工作目标，重点要抓好以下工作：

（一）深入实施脱贫攻坚战，加快全面小康进程。

大力推进扶贫基础设施建设。加强贫困村屯级道路、水利、网络、电网、贫困户危房等基础设施建设。加快实施20户以上自然村（屯）道路硬化和饮水安全进村入户工程，基本实现行政村宽带信息网络全覆盖，全面解决贫困村用电难问题，完成危房改造2.13万户。推进易地扶贫移民搬迁工程，实施易地移民搬迁3.2万户13.2万人。

强化精准脱贫措施。加快推进扶贫产业综合示范区建设。大力推动生态扶贫、旅游扶贫、光伏扶贫、电商扶贫和边贸扶贫。推进教育精准脱贫，落实贫困户子女从学前教育到高等教育各阶段学生资助政策，实施“农村贫困家庭高校毕业生精准就业帮扶行动”。实施好健康扶贫工程“五个一行动”，建立贫困户健康档案并完善数据库，做好大病保险、医疗救助、商业健康保险等制度衔接。继续抓好全国政策性金融扶贫实验示范区建设，推动一批扶贫项目落地，加快发展扶贫小额信贷，提高信贷资金使用效益。强力推进边境地区、水库移民区等特殊区域扶贫。

全面推进深圳百色扶贫协作。深化与深圳市在产业扶贫、教育扶贫、金融扶贫、劳务输出、易地搬迁、基础设施建设、干部培训交流、生态建设等领域帮扶合作，建立完善帮扶协作项目库，加快启动百东新区深圳异地扶贫搬迁小镇、深百协作产业园建设，共建百色国家农业高新区。推动“两后生”职业技能培训合作，大力开展劳务协作，分批组织贫困劳动力赴深圳务工。确保实现一批项目落地，一批贫困人口脱贫。进一步发挥社会和企业的帮扶作用。

切实增强脱贫工作实效。严格执行党政主要领导脱贫攻坚责任制。完善脱贫攻坚督查工作机制，抓好县乡扶贫开发成效考核和实绩考核。进一步完善“三双”精准扶贫工作机制，抓好干部结对帮扶。做好精准识别建档立卡数据库的动态管理，加强对脱贫户的动态跟踪帮扶。加强扶贫领域的监督执纪问责，完善扶贫资金违规使用责任追究制度，保障扶贫资金安全运行。

（二）加快产业转型升级，培育经济增长新动能。

加快推进“铝二次创业”。继续推进煤电铝一体化，建立电解铝与氧化铝的价格联动机制，确保自治区确定的铝“二次创业”战略部署全面实施。积极推进电源建设，启动区域电网二期工程，引滇电、黔电入百，建设百色至贵州兴义输变电线路工程。加强与广西电网合作，完善政府用电安全联席会议制度，确保百色区域电网安全运行。支持百矿集团参与区内外煤源并购，收购苏源煤电铝项目。加快百矿集团德保、田林、隆林3个煤电铝一体化项目建设。支持百矿集团拉长产业链，实现年产1万吨精铝项目竣工投产，推动百矿集团成为全产业链的煤电铝一体化龙头企业。推动百矿集团、广西信发、苏源和银海铝业现有电解铝产能投产达产。实现广西华磊年产40万吨电解铝及3台35万千瓦自备电厂项目竣工投产。推动曾氏集团年产200万吨氧化铝、100万吨电解铝及80万吨铝材加工项目落地。推进铝精深加工，加快德保年产10万吨铝精深加工和5万吨铝合金锭加工项目建设，加快推进中车集团铝深加工、正大集团1000万套铝轮毂项目尽快落地。加强与上海交大等科研院所合作，加快精铝、高纯铝以及航空航天、船舶、轨道交通用铝、锻造铝轮毂等铝精深加工产品研发，推动铝产业全产业链发展。力争铝产业产值突破千亿元大关，建成百色第一个千亿元产业。

推动传统工业提质增效。推进甘蔗综合利用、拉长产业链，推进制糖企业兼并重组。实施锰、铜产业技术改造，加快锰加工企业整合重组，建设生态锰产业示范基地。抓好田东石化总厂项目技改。推动化工产业转型升级，建设锦江集团二期30万吨烧碱项目，力争锦江集团20万吨甲烷氯化物技改系列项目建成投产。统筹抓好建材、造纸及农林产品深加工。推动百色水务公司等企业上市。

大力发展现代特色农业。新创建各类现代特色农业（核心）示范区50个以上。探索智慧农业发展模式。推进百万亩芒果、百万亩柑桔、百万亩桑蚕和“田七回家”工程，完成“双高”糖料蔗基地建设。完成新种水果15万亩，总面积突破270万亩。新种桑园5万亩，总面积突破40万亩。抓好40个油茶示范区建设，新种油茶9万亩，低产改造15万亩。推进蔬菜标准化生产，面积稳定在180万亩以上。推进茶园低产改造。加快发展现代生态养殖，做稳生猪产业、做大牛羊产业、做优林下经济、做强现代渔业，创建10个特色养殖产业示范区（园），100个“一村一品”特色养殖增收示范村。稳步推进百万亩油茶等“5个百万亩”特色林业基地建设，扎实推进县（市、区）林产品加工园区建设，加快组建百色林业集团。筹建右江河谷农产品质量安全检测中心。做优做强百色芒果、百色红茶、百林鸡等特色农产品，推动建设一批无公害、绿色和有机农产品生产基地。推进百色山茶油、百色色拉油等新品牌创建，力争全年新增3个以上地理标志保护产品。加强新型农业经营主体培育，筹组农业开发投资公司，力争新增市级龙头企业10家、农业专业合作社100家、家庭农场100家以上。举办好第十二届世界芒果大会。

提升发展现代服务业。加快发展全域旅游。启动滇桂黔（百色）国际旅游集散中心项目建设。加快推进巴马长寿养生国际旅游区基础设施。招商建设一批五星级酒店。继续推进田阳田州古城、凌云浩坤湖、乐业大石围天坑群景区等重大旅游项目建设。抓好广西特色旅游名县、休闲农业与乡村旅游示范点创建。推动中越边关风情旅游带，边境旅游试验区、跨境旅游合作区建设。加快推进百色起义纪念园等景区创5A工程，抓好一批A级旅游景区基础设施改造。积极培育乡村旅游、休闲养生旅游、旅游+文化体育等新业态。组建百色文化旅游发展有限公司。加强景区服务管理。加快建设现代服务业聚集区，推进百色义乌国际商贸物流城、亿丰百色国际商博城等商贸物流项目。加快中国-东盟农产品交易中心建设，推动“百色一号”专列正常运营，力争每月运行3列。构建东盟国家农产品集散大平台。推动金融产品和服务创新，筹建百色银行。

积极培育新经济新产业。实施好电子商务进农村示范工程，加快电商、物流、快递等配套行业建设，推动电商平台在我市扩大营运规模。加快科技创新、引进吸收、应用推广，推进铝基新材料研发，提升赤泥综合利用。加快推进碳酸钙产业基地建设，打造新的百亿元产业。积极发展大数据、云计算、电子信息、生物医药、节能环保等产业。加快发展健康产业，出台养老养生健康服务业发展规划，加快建设鼎盛和泰养生文化园，推进百色养生文化综合体项目前期工作。研究推进百色国家级医养结合试点建设。加快茶酒项目落地生产。

（三）深入实施交通等基础设施建设，夯实发展承载能力。

推动建成南昆铁路新增二线百色段，推进黄桶至百色、靖西至龙邦、云桂沿边铁路，百色至河池、柳州铁路，百色至兴义高速铁路等项目前期工作。建成靖西至龙邦高速公路，加快推进河池至百色、乐业至百色高速公路建设，力争开工建设田林至西林高速公路。抓好一批国省干线路网提级改造，畅通主干路网。完成百色机场盲降系统建设，加大航线培育力度，落实开通百色至北京航线。做好百色机场迁建和乐业通用机场选址工作。推进百色港码头项目建设，实现祥周、二塘等作业区投入运营。加大航运市场培育，发展集装箱班轮，实现右江黄金水道航运常态化。完成百色水库灌区项目前期工作，年底前实现单体工程开工建设。完成13座抗旱小型水库主体工程。完成澄碧湖等水库除险加固工程。加快一批中型水库项目前期工作。启动一批新扩建小型水库、备用水源等水利项目前期工作。

（四）加快新型城镇化建设，统筹城乡协调发展。力争常住人口城镇化率提升到37.7%以上。

积极推进新型城镇化。抓好百东新区、百色工业园区、新山生态型铝产业示范园区创建国家产城融合试点建设，争创国家产城融合试点市。加快右江-田阳一体化进程，建成百色大道四塘-田阳段，启动田阳-平果段建设前期工作。加快推进百东新区市政基础设施建设，加快推进田阳撤县设区、平果撤县设市工作。推进平果县、靖西市国家新型城镇化综合试点城市建设。加快建设旅游小镇、边贸小镇、欢乐小镇、民族风情小镇等一批特色小镇。加快农业转移人口落户城镇，力争实现8万人以上进城落户。推进海绵城市、地下综合管廊、智慧城市建设和城中村改造等工作，开展城市修补和生态修复“双修”工程，加快完善各新建城区、扶贫移民搬迁小镇等配套功能。进一步理顺和规范规划、建设、管理关系，完成《百色市城市总体规划》修编，有序推进城镇总体规划、土地利用规划、百色市中心城区人民防空工程和经济社会发展规划等“多规合一”。做好历史文化名城保护规划、城市地下空间开发利用总体规划编制，抓好历史文化名城、中国篆刻名城创建工作。开展城市建成区违法建设专项联合治理五年行动。继续实施中心城区功能提升服务“十大工程”。做好国家卫生城市复审迎检工作。推进“广西宜居城市”创建活动。继续深入实施城乡风貌改造和“美丽百色·宜居乡村”建设工程。

壮大发展县域经济。右江河谷三县一区着力做大做强现有产业，实现县域经济率先发展，打造我市经济社会发展龙头板块。南部山区三县发挥后发优势，突出铝、锰、旅游、边贸等产业，奋力快速崛起，打造我市经济发展的潜力板块。北部山区五县发挥山水民族文化资源优势，充分释放生态涵养功能，突出发展特色生态农业、旅游、林业、中医药、健康养生等产业，打造我市经济发展的绿色板块。加强县域经济发展考核，突出差异化考核评价机制。

（五）深入推进改革创新，加快形成发展新优势。

深化供给侧结构性改革。持续推动“三去一降一补”各项政策的落地实施。有序引导“僵尸企业”平稳退出，扎实推进石化、水泥、冶金、建材等行业的整合重组。继续支持新型城镇化农民进城购房、棚户区改造货币化安置等政策落实，加快培育住房租赁市场，确保商品房库存保持在合理水平。健全完善政府性债务管理体系，加大政府存量债务置换力度。拓宽融资渠道，优化信贷投向和期限，提高企业股权、债券等直接融资比重。继续完善提升区域电网一期工程运营水平，积极推进电力直接交易，提高电力保障力度。完善左右江振兴发展基金、扶贫开发基金和城镇化基金募集、投资、运营管理。完善降成本“43条”措施，减免一批涉企行政事业性收费项目，落实好“营改增”、困难企业延期缴纳税款、小微企业税收优惠等政策，切实减轻企业运行成本。继续实施“五降一缓三补贴”政策，帮助企业减压增效。加大政策、资金扶持，进一步完善基础设施，补齐企业产业链和精深加工等短板。

深化重点领域改革。继续深化“放管服”改革，清理规范行政审批及中介服务事项，完成政府权力清单和责任清单公开，推进项目审批一站式服务。推进工商登记制度改革，实施全程电子化登记，深化“先照后证”改革。着力构建事中事后监管新机制，全面推进“双随机、一公开”监管改革。加快出台国有企业改革实施意见，优化国有企业资产配置。推进经营性国有资产集中统一监管，推动煤电铝、煤电锰等国有资产重组整合。完善国有企业领导人员薪酬体系，探索实行企业负责人绩效考评奖金与企业规模经营效益挂钩。稳妥推进国有企业“三供一业”移交社会管理，推动百色通用机械厂、氮肥厂改制。推进农村综合改革，基本完成农村承包土地经营权确权颁证试点，抓好农村宅基地确权登记试点、农村土地流转、集体林权制度配套改革，加快林权流转平台建设。基本完成农村土地确权登记颁证工作，基本完成全市22家国有林场改革，全面停止国有林场天然林商业性采伐。加快小型水利工程管理体制改革。推进供销社综合改革。继续深化医药卫生体制改革，推进城市公立医院改革。加快完善不动产统一登记制度，推进不动产登记信息化建设。加快推进金融改革创新，开展产城、产融融合试点，探索组建百色金融控股集团、设立百色产业并购基金。深化沿边金融综合改革，推进跨境金融合作。

大力推进创新驱动发展。设立百色创新发展基金。推进实施百色“蓝火计划”。搭建科技成果交易服务平台。创建铝“二次创业”成果转化中心。加快中国（百色）-东盟农业科技研发中心、国家林木种质资源库、铝合金材料研究中心、铝产业研究院、铝产品工程技术研究中心、新材料研究院等科研机构建设，申报3家自治区级工程技术研究中心、3家创新型企业。打造百色铝金属产品检验检测人才小高地。实施“质量强市”战略，打造一批广西名牌、服务业品牌、优质工程、著名商标、老字号。全面推进大众创业万众创新。

加快创建百色国家农业高新区。引进国内外创新团队，搭建国家石漠化综合治理工程技术研究中心平台，推动三次产业深度融合。建设现代农业科技中试孵化基地，设置农产品精深加工区、现代小型农机装备产业园、生物医药产业园、健康养生产业园，构建高技术产业聚集平台。建设中国-东盟（广西百色）会展中心、百万亩芒果实训基地，搭建产城融合平台。

（六）促进投资和消费，积极扩大有效需求。

扩大有效投资。实施重大项目建设攻坚行动，加大项目前期经费投入，扎实做好项目前期工作。抓好左右江革命老区三年行动计划30个项目、珠江-西江经济带基础设施大会战10个项目、巴马长寿养生国际旅游区基础设施23个项目建设。策划包装公共交通、养老服务、跨境电子商务、工业园区、教育卫生、文化体育等领域重大项目，统筹推进自治区层面重大项目50个以上，市层面重大项目500个以上。实施好中央预算内投资项目，争取更多专项建设基金项目和政府债券项目。做好引导和服务，实施好百东新区纬六路、市社会管理综合运用云平台、百隆高速永乐出口连接线、市高铁站站前服务广场、中国-东盟现代农业展览馆等5个PPP重点项目,继续推出一批PPP项目。发挥各级融资平台功能，支持开展股权投资，大力促进民间投资。

促进消费提质升级。继续开展“广西特产行销全国”百色芒果推介活动，扎实做好特色农产品网络展销、农超对接等促销工作，完善农产品购销体系。抓好各县（市、区）特色节庆活动，促进节假日消费。开展汽车消费促进行动，支持建设二手车交易市场。继续实施“个转企、小上限”工程，力争培育限上企业30家以上。推动宽带乡村示范工程，改善农村地区消费基础设施，支持电商和物流企业向乡镇农村延伸，挖掘城镇、乡村消费潜力。推进服务消费提质扩容，挖掘旅游、文化、体育、健康、养老、教育培训等消费潜力。开展“诚信经营、放心消费”创建活动，严打假冒伪劣、侵权宰客行为，优化消费环境。

（七）扩大开放合作，加快门户经济发展。

加快推进沿边开发开放。完成沿边对外开发开放总体规划，实施口岸基础设施建设攻坚年行动，彻底改善口岸区域的水、电、路、网等基础设施建设，将所在乡镇纳入基础设施建设行动，推动边境乡镇和口岸建设融合发展，打造边陲特色小镇。加快推进龙邦进境水果、粮食指定口岸、国检试验区建设，力争年内通过验收并通关营业。加快那坡弄平互市贸易区建设，开通新兴边民互市点。推进互市产品落地加工。探索建设东盟大牲畜产品进境指定口岸试验区。

加快推进区域合作。以推进靖西万生隆国际商贸物流中心建设为重点，加快建设龙邦-茶岭跨境经济合作区、平孟综合物流园区，推进中国-柬埔寨合作产业园、中国-印尼“两国双园”百色（田东）产业园建设，创建中国-东盟农产品自由贸易示范区，着力在资源开发利用、产能合作、加工贸易、仓储物流等合作取得新突破。加快建设百色-文山、百色-黔西南跨省（区）经济合作园区，深化煤电铝一体化、农业、旅游、石漠化防治生态功能区的合作。

促进外贸提质升级。加快建立外贸工作运转协调机制，研究制定市、县层面的政策制度、预测预警机制。设立对外贸易发展促进资金，支持靖西、那坡出台边境贸易优惠政策，促进对外贸易企业发展。加强与中国出口信用保险公司合作。支持培育对外贸易龙头企业，积极引进承接东部产业转移的加工贸易载体，支持铝及其加工产品等优势产品出口。引导企业加入“一达通” 等电商平台，引导铝工业、小型机械、特色农产品等企业利用电子商务平台拓展进出口业务。探索在越南等东盟国家设立百色铝材产品东盟展示展销中心。

强化精准招商。加大铝精深加工、现代特色农业、现代服务业和新兴产业招商引资。重点在旅游、高新技术产业、总部经济、大数据、电子信息、生物医药、节能环保、健康养生养老等方面，研究出台有针对性的招商优惠政策，完善招商引资协调推进机制，加快项目落地实施。参加广交会、专题展等招商活动，用好“加工贸易倍增计划”平台，组织策划在深圳开展铝产业专题招商活动。力争实现区外招商引资到位资金420亿元。

（八）加强生态建设，推进绿色发展。

加强生态基础建设。实施植树造林、石漠化治理和新一轮退耕还林工程，完成植树造林50万亩，实现森林覆盖率67.5%。加强生态保护红线管控，加大林地、国家野生保护动植物、自然保护区、森林公园、湿地公园、风景名胜区及生态公益林的保护力度。加强耕地、地质遗迹保护，加大土地整治，实行耕地“占优补优”。巩固提升清洁乡村、生态乡村建设成果，稳步推进宜居乡村建设。力争成功创建“国家森林城市”。

加强环境治理和保护。严格执行大气十条、水十条、土十条，落实“河长制”。开展第二次污染源普查，建立健全重点污染源档案、信息数据库和环境统计平台。狠抓城市扬尘、工业污染、移动源污染、秸秆垃圾焚烧等污染源防控，PM10、PM2.5浓度和主要污染物排放量控制在自治区下达指标以内。加强噪声污染治理。严格落实水污染防治行动计划，加强城市生活污水、工业污水、乡镇污水处理设施建设，提高污水收集处理率。继续加强城市黑臭水体整治，全市要消除60%以上。抓好右江流域污染治理和饮用水源保护。突出治理澄碧河水质下降及下雷河污染物超标问题，确保水质全面达标。建设土壤环境质量监测网络，加快推进重金属污染防治项目和危险化学品管理。推进土壤污染治理与修复试点示范。继续推进农村环境连片综合整治项目建设。大力推广绿色建筑，推进全国建筑节能试点城市项目建设。继续抓好中央环境督察组反馈问题的后续整改。加强环境监管和生态保护执法，严厉打击各种环境违法行为。

加快发展生态经济。加快铝、冶金、石化等传统产业生态化改造。加快推进园区循环化改造、资源综合利用项目，把百色工业园区、田东石化工业园区、百色新山铝产业示范园、平果工业园区打造成为循环经济、生态工业示范园区。实施节能技术改造和清洁生产示范项目建设，促进工业节能降耗水平提升。加快发展生态农业，减少化肥农药使用量，推广生态养殖，发展生态林业，切实保护农业生态环境。推动健康养生、有机食品、生态旅游等产业加快发展。

（九）大力发展民生和社会事业，增强群众幸福感。

做好就业创业工作。落实和完善积极的就业政策，重点抓好高校毕业生和就业困难人员就业工作。深入开展“百千万”结对帮扶精准就业扶贫工作。抓好农村劳动力转移就业和返乡创业工作。加大贫困家庭职业技能培训力度。

健全社会保障体系。推进农村低保制度与扶贫政策有效衔接，提高低保政策兜底脱贫水平，提高特困人员救助水平。继续做好机关事业单位养老保险制度并轨改革。整合城镇居民医保和新农合资源，建立健全城乡居民医疗保险制度。继续做好80周岁以上高龄老年人津贴发放。实施“全民参保登记计划”，落实和完善社保衔接转续政策，实现全员动态管理。

大力发展教育事业。继续实施中心城区教育基础设施建设攻坚战，狠抓教育工程项目建设。继续实施学前教育行动计划，扩大公办幼儿园规模，出台政策鼓励引导民间资本发展学前教育，力争学前三年毛入园率达到85%以上。做好县域义务教育均衡发展督导评估迎检工作，确保平果县、凌云县、乐业县通过国家级评估认定，确保全市九年义务教育巩固率达到92.5%。全面实施义务教育学区制管理改革。抓好一批普通高中学校新建、改扩建工程。进一步深化职业教育综合改革，加大职业教育扶贫助学力度，加快普及高中阶段教育步伐。加快推进教育信息化建设。加强教师队伍建设。推进右江民族医学院、百色学院、百色职业学院百东新校区建设。

突出抓好卫生计生工作。推进医疗改革，做好分级诊疗和医联体工作，加强医疗卫生城乡一体化建设，提高基层医疗服务能力。深化实施城乡居民大病保险工作，推进新农合大病保险即时结算“一站式”服务，严格控制医疗费用不合理增长。加快实施医疗卫生重点项目建设，加强卫生人才队伍培养，强化对基层乡村医生的教育培训，加大全科医生培养力度。落实全面两孩政策，推进计划生育服务管理改革。加大重大传染病防控力度。继续推动中医药壮瑶医药事业发展。推进健康扶贫。推进“健康百色”建设。

提升文化体育服务水平。继续推进国家级百色壮族文化生态保护区建设。推进文化产业项目申报，推进村级公共文化服务中心、非遗传习基地等文化项目建设。支持文艺精品创作和演出，加强新闻出版、广播电视和文化市场管理工作。改善城乡体育健身设施，大力发展群众体育，推进全民健身运动。加快推进百色体育中心二期、“三馆一中心”、百东新区体育休闲公园项目建设，推进妇女儿童活动中心、广播电视播控中心等一批公共服务项目。做好广西第十四届运动会筹备和备战工作，加快培养竞技体育人才。加快足球进校园工作。大力发展体育旅游休闲产业和体育竞赛表演业，打造一批具有影响力的体育旅游品牌赛事，促进体育与旅游、文化、健康产业融合发展。

继续实施为民办实事工程。实施社保、健康、教育、水利、安居、农补、生态、文化、扶贫、交通等为民办实事工程项目。

加强和创新社会治理。推进城乡社区建设，开展农村社区建设试点单位创建活动，加强乡村治理法治化，积极开展社区协商，全面推行社区公共服务事项准入制度。加强诚信建设。推进应急、安全生产、食品药品安全建设。深入推进法治百色、平安百色建设，构建立体化现代化社会治安防控体系。严厉打击黑恶势力、涉枪涉爆、毒品犯罪、网络犯罪、非法集资、盗抢骗、传销等违法行为，扎实开展反恐防暴工作。深化信访工作制度改革。建立健全公共法律服务体系，狠抓执法规范化建设。

支持部队和国防建设。加强和改进新形势下民族工作，积极推进迎接自治区成立60周年大庆筹备工作。充分发挥工会、共青团、妇联、残联、红十字会、科协、文联等群团组织作用。继续做好宗教、侨务、调处、统计、气象、地震、测绘、人事、编制、档案、老龄、地方志、决策咨询、哲学社会科学等工作。

加强政府自身建设，更好发挥政府作用。深入开展投资环境专项整治活动。推行“互联网+政务服务”，推动决策、执行、管理、服务和结果“五公开”，扩大群众参与公共政策制定、执行和监督渠道，做好重大政策解读和政务舆情回应工作。坚持政府常务会学法制度。完善政府立法体制机制，加大城市建设与管理、环境保护、历史文化保护等方面的立法工作。推进城市建设管理综合执法体制改革，推行跨部门综合执法，推动乡镇延伸综合行政执法，实现“四所合一”、“多所合一”。坚持规范文明执法，依法化解矛盾纠纷，营造良好的法制环境。坚持政府常务会集体决策制度，健全公众参与、专家论证、风险评估、合法性审查、集体讨论决定等依法决策机制。建立行政机关内部重大决策合法性审查机制。严格行政决策过错责任追究，充分发挥政府法律顾问作用。自觉接受人大依法监督和政协民主监督，认真听取人大代表、政协委员及各民主党派、工商联、无党派人士、各人民团体的意见建议，办好人大代表议案建议和政协提案。巩固“两学一做”学习教育成果，坚持查纠“四风”，推进作风建设规范化、常态化、长效化。坚决落实中央八项规定精神和国务院“约法三章”，坚持有腐必反、有贪必肃。不断完善审计监督全覆盖。建立健全督促检查工作机制，抓好决策部署、重大政策、重大工程和重大项目的推进落实。
